# KRT13 promotes stemness and drives metastasis in breast cancer through a plakoglobin/c-Myc signaling pathway

**DOI:** 10.1186/s13058-022-01502-6

**Published:** 2022-01-25

**Authors:** Lijuan Yin, Qinlong Li, Stefan Mrdenovic, Gina Chia-Yi Chu, Boyang Jason Wu, Hong Bu, Peng Duan, Jayoung Kim, Sungyong You, Michael S. Lewis, Gangning Liang, Ruoxiang Wang, Haiyen E. Zhau, Leland W. K. Chung

**Affiliations:** 1 Department of Pathology, West China Hospital, Sichuan University, Chengdu, Sichuan China; 2 Uro-Oncology Research Program, Samuel Oschin Comprehensive Cancer Institute, Department of Medicine, Cedars-Sinai Medical Center, 8750 Beverly Boulevard, Atrium 105, Los Angeles, CA 90048 USA; 3 Division of Cancer Biology and Therapeutics, Departments of Surgery and Biomedical Sciences, Samuel Oschin Comprehensive Cancer Institute, Cedars-Sinai Medical Center, Los Angeles, CA USA; 4 Department of Pathology, VA Greater Los Angeles Healthcare System, Los Angeles, CA USA; 5 Department of Urology, University of Southern California Keck School of Medicine, Los Angeles, CA USA

**Keywords:** Breast cancer, Metastasis, KRT13, Plakoglobin, γ-Catenin, c-Myc

## Abstract

**Background:**

Keratins (KRTs) are intermediate filament proteins that interact with multiple regulatory proteins to initiate signaling cascades. Keratin 13 (KRT13) plays an important role in breast cancer progression and metastasis. The objective of this study is to elucidate the mechanism by which KRT13 promotes breast cancer growth and metastasis.

**Methods:**

The function and mechanisms of KRT13 in breast cancer progression and metastasis were assessed by overexpression and knockdown followed by examination of altered behaviors in breast cancer cells and in xenograft tumor formation in mouse mammary fat pad. Human breast cancer specimens were examined by immunohistochemistry and multiplexed quantum dot labeling analysis to correlate KRT13 expression to breast cancer progression and metastasis.

**Results:**

KRT13-overexpressing MCF7 cells displayed increased proliferation, invasion, migration and in vivo tumor growth and metastasis to bone and lung. Conversely, KRT13 knockdown inhibited the aggressive behaviors of HCC1954 cells. At the molecular level, KRT13 directly interacted with plakoglobin (PG, γ-catenin) to form complexes with desmoplakin (DSP). This complex interfered with PG expression and nuclear translocation and abrogated PG-mediated suppression of c-Myc expression, while the KRT13/PG/c-Myc signaling pathway increased epithelial to mesenchymal transition and stem cell-like phenotype. KRT13 expression in 58 human breast cancer tissues was up-regulated especially at the invasive front and in metastatic specimens (12/18) (*p* < 0.05). KRT13 up-regulation in primary breast cancer was associated with decreased overall patient survival.

**Conclusions:**

This study reveals that KRT13 promotes breast cancer cell growth and metastasis via a plakoglobin/c-Myc pathway. Our findings reveal a potential novel pathway for therapeutic targeting of breast cancer progression and metastasis.

**Supplementary Information:**

The online version contains supplementary material available at 10.1186/s13058-022-01502-6.

## Background

Breast cancer is the leading cause of cancer-related death among women worldwide. It is a heterogeneous disease with divergent molecular alterations and cellular changes. Distant metastasis is the final stage of breast cancer progression. The pathobiological features and the exact mechanism are far from being completely understood.

Keratins (KRTs) are considered structural proteins ranging in sizes from 40 to 76 kDa. They are the major component of intermediate filaments (IFs) in the intracytoplasmic cytoskeleton of epithelial and endothelial cells. IFs insert into the electron-dense desmosomal plaques and connect to other IFs to provide tensile strength to cells. Aberrant expression in various cancers makes KRTs useful as biomarkers for differential diagnoses and metastatic status. Recent studies suggest that KRTs in cancer cells are not only epithelial marker proteins but are also mediators capable of interacting with a range of proteins to regulate signaling networks associated with cell death, survival, proliferation, migration, invasion and metastasis [1–3].

KRT13, a 54 kDa type I keratin that often pairs with type II keratin 4, is found in the suprabasal layers of non-cornified stratified squamous epithelia of the oral cavity, tonsils, larynx, esophagus, lower genital tract and transitional urothelium [4, 5], where KRT13 is a major component of the basal and intermediate cells and enriched in prostatic tubule-initiating cells [6, 7]. KRT13 expression can be regulated by factors including calcium, nuclear receptor ligands such as retinoids and 1α, 25-dihydroxyvitamin D3, and estrogens or selective estrogen receptor modulators [8, 9]. Point mutation of KRT13 was shown to be correlated with autosomal dominant disorder white sponge nevus [10]. Dysregulated KRT13 expression was found in carcinomas of the tongue, head and neck, uterine cervix, mouth, ovary, breast, bladder cancer, esophageal cancer, and prostate cancer [11, 12].

KRT intermediate filaments are involved in the translation of environmental cues into modifications of gene expression [13]. KRT is physically linked to nuclear membrane and desmosomes, where plakoglobin (PG) is a multifunctional protein. Also known as γ-catenin, PG is a paralog of β-catenin, both belonging to the Armadillo protein family. As a major component of the adherens junctions and desmosomes, PG coordinates with desmoplakin (DSP) to anchor intermediate filaments to desmosomes [13]. PG also participates in cell signaling regulation, but with an antagonistic effect to β-catenin. While β-catenin has a well-defined oncogenic potential through the Wnt signaling pathway, PG exhibits tumor/metastasis suppressor activity [14]. Recent evidence suggests that PG in the cytoplasm and nucleus can regulate tumor progression and metastasis [15]. PG may implement its regulatory function by competing with β-catenin, interacting with intracellular proteins, or sequestering transcription factors [16, 17].

We previously reported that KRT13 promotes prostate cancer bone and brain metastases through RANKL-independent pathways [18] by an undefined mechanism. In this study, we explored the mechanism by which KRT13 promotes breast cancer progression and metastasis. Overexpression of KRT13 led to increased breast cancer cell proliferation, migration and invasion in vitro, and tumor formation and metastasis in vivo, while knockdown of KRT13 resulted in alleviating effects in vitro and in vivo. Importantly, we report for the first time that KRT13 directly interacts with PG/DSP complexes and alters the expression and nuclear translocation of PG, thus modulating downstream c-Myc-signaling. Our findings provide a potential new therapeutic target in breast cancer progression and metastasis.

## Methods

### Cell culture

Human breast cancer cell lines MCF7 (indolent, low endogenous KRT13 expression) and HCC1954 cells (aggressive, high endogenous KRT13 expression) were provided by Dr. X. J. Cui at Cedars-Sinai Medical Center (CSMC). MCF7 cells were maintained in DMEM (ThermoFisher Scientific, Waltham, MA) and HCC1954 was maintained in RPMI1640 (ThermoFisher Scientific). Both culture media were supplemented with 10% fetal bovine serum (Atlanta Biologicals, Flowery Branch, GA), 100 IU/ml penicillin, and 100 μg/ml streptomycin. All cells were maintained at 37 °C in a humidified atmosphere with 5% CO_2_.

### Plasmids, siRNA transfection and viral transduction

For KRT13 overexpression, a cDNA containing the full-length open reading frame of human KRT13 (NM_153490) was subcloned into pLVX-AcGFP1-N1 (pLV) (Clontech, CA) by introducing EcoR I and BamH I sites. The construct of pLVX-AcGFP1-N1-KRT13 (pLV-KRT13) was confirmed by DNA sequencing. The plasmid DNA was transfected to the 293 T cells to produce lentiviral particles, following the manufacturer’s instructions (System Biosciences, Mountain View, CA). MCF7 cells were transduced with the lentivirus and selected with puromycin (2 µg/ml for 2 weeks). For shRNA-mediated knockdown, non-targeting control or KRT13 shRNA lentiviral particles (Santa Cruz Biotechnology, Dallas, Texas) were used to infect the HCC1954 cells. The plasmid pcDNA3-c-Myc (Addgene, Cambridge, MA) was used to generate c-Myc-overexpressing cells. To silence c-Myc, cells were transfected with c-Myc siRNA (Santa Cruz Biotechnology) using Lipofectamine 2000 transfection reagent (Invitrogen, Carlsbad, CA).

### Cell proliferation and behavioral assays

To determine growth rates, breast cancer cells were seeded on 24-well plates for 5 days, and cell numbers from triplicate wells were counted daily with a TC20 automatic cell counter (Bio-Rad, Hercules, CA). Cancer cell migration was examined in triplicate transwells (8 μm pore size) coated with collagen I. For invasion assays, transwells were coated with collagen I, and each well was overlaid with growth factor reduced Matrigel (BD Biosciences, San Jose, CA) as previously described [19]. After incubation at 37 °C for 24–48 h, cells on the upper surface of the filters were removed by cotton swab, and cells that had invaded and migrated to the lower surface were fixed with 10% formaldehyde and stained with 0.5% crystal violet. After washing, stain was eluted with Sorensen’s solution. The absorbance of each well was measured at 590 nm.

### Cell fractionation and western blot analysis

Cytoplasmic and nuclear extracts or whole cell lysates were prepared with NE-PER nuclear and cytoplasmic extraction reagents (ThermoFisher Scientific). Western blot analysis was performed as previously described [19]. The primary antibodies used were to KRT13 (EPR3671, Abcam, Cambridge, UK or B-2, Santa Cruz Biotechnology), CD44 (156-3C11, Cell Signaling, Danvers, MA), c-Myc (D84C12, Cell Signaling), ALDH1A1 (B-5, Santa Cruz Biotechnology), Nanog (5A10, Santa Cruz Biotechnology), PG (A-6, Santa Cruz Biotechnology), DSP [EPR4383(2), Abcam], Actin (AC-15, Santa Cruz Biotechnology) and Lamin B (C-20, Santa Cruz Biotechnology). Immunoblots were subjected to morphometric analysis by Image Studio Software (LI-COR, Lincoln, NE).

### Quantitative reverse transcription and polymerase chain reaction (qRT-PCR) and RNA sequencing (RNA-seq) analyses

Total RNA was isolated using the RNeasy Mini Kit (Qiagen, Hilden, Germany). For RNA-seq analyses, duplicate samples were submitted to University of California Los Angeles (UCLA) Clinical Microarray Core for RNA-seq analysis. The RNA-seq data were first subjected to differentially gene expression with the DESeq2 program. Gene set enrichment analysis was then performed. For correlation study, RNA-seq gene expression pattern was compared to breast cancer subtypes of The Cancer Genome Atlas (http://cancergenome.nih.gov). To assess correlation between breast cancer subtypes and KRT13 overexpression cells, we compared expression centroids of the five defined breast cancer subtypes [20, 21] with the KRT13 overexpressing MCF7 cells. Unsupervised clustering with Pearson’s correlation was performed to display the similarity in the heatmap with dendrogram. To analyze specific gene expression, qRT-PCR was conducted with experimental settings as we previously reported. Sequences of the primer pairs are listed in Additional file 1: Table S1.

### Clinical specimens

Formalin-fixed and paraffin-embedded tissue specimens including 41 primary, 11 bone and 10 brain metastatic breast cancer tissues were obtained from West China Hospital, Chengdu, China. Handling of tissue specimens conformed to the policies and practices of the institutions. The use of the specimens was approved by the Institutional Research Board of the CSMC (IRB: Pro00054328).

### Immunohistochemical analyses

Our immunohistochemical (IHC) staining protocol is previously published [22]. Primary antibodies to KRT13 (ERP3671, Abcam), PG (A-6, Santa Cruz Biotechnology), and c-Myc (D84C12, Cell Signaling) were used. For this study, images of stained sections were scanned with a Keyence BZ-X710 microscope (Itasca, IL). The stains were scored with combined percentage of positive cells and intensity as reported [23].

For immunofluorescence staining, sections were stained with Alexa Fluor 594‐conjugated goat anti‐rabbit IgG (H + L) or Alexa Fluor 488‐conjugated goat anti‐mouse IgG (H + L) secondary antibodies (ThermFisher Scientific). Sections were further counterstained with 4′,6-diamidino-2-phenylindole (DAPI) in mounting medium (Vector Laboratories, Burlingame, CA) and examined by Zeiss Axio Observer Z1 fluorescence microscope.

Multiplexed quantum dot labeling (mQDL) analysis was performed as we previously reported [24]. Tissue sections were sequentially incubated with antibodies to KRT13, PG and c-Myc. Areas of interest were defined with manual segmentation by a pathologist (M. S. Lewis).

### Flow cytometry

Flow cytometric analyses were conducted as described previously [23]. Cultured cells at 80% confluence were prepared in single cell suspension and fixed in 4% formaldehyde, washed in phosphate buffered saline (PBS), resuspended in PBS containing 1% BSA, and incubated with APC-conjugated anti-CD44 (IM7, Biolegend) and PE-conjugated anti-CD24 (ML5, Biolegend) antibodies at 4 °C for 30 min. Matched APC- or PE- conjugated isotypes were used as negative controls.

### Mammosphere assay

Cells were plated in ultra-low attachment 96-well plates (Corning Inc., Corning, NY) in serum-free DMEM/F12 medium (ThermoFisher Scientific) supplemented with 20 ng/ml epidermal growth factor (ThermoFisher Scientific), 10 ng/ml basic fibroblast growth factor (Pepro Tech, Rocky Hill, NJ), 5 µg/ml insulin (VWR, Radnor, PA), 1 × B-27 supplement (ThermoFisher Scientific) and 0.4% bovine serum albumin (Sigma, St. Louis, MO). For each experimental group, 500 cells in 100 µl medium were plated to each of 10 wells. After cultured for a week [25], spheres were counted and documented with phase contrast microscopy. Each study was repeated for three times.

### Co-immunoprecipitation (co-IP) analysis

Cell lysates (50 µg) were precleared by incubating with 1 µg rabbit/mouse control IgG in 20 µl Protein A/G PLUS-Agarose suspension (Santa Cruz Biotechnology) at 4 °C for 1 h. After centrifugation, the supernatant was incubated with primary antibodies at 4 °C overnight, and then with 20 µl of Protein A/G PLUS-Agarose at 4 °C for 2 h. Immunoprecipitates were collected and rinsed four times with cell lysis buffer. Half of each sample was submitted to the Mass Spectrometry and Biomarker Discovery core at CSMC for interactome analysis, and the remaining half was used to verify the interactome results by western blot.

### In vivo* experiments*

All animal procedures were performed according to a protocol approved by the Institutional Animal Care and Use Committee (IACUC) of the CSMC as previously described [18, 19]. MCF7-KRT13 or HCC1954-shKRT13 cells were tagged for luciferase reporter expression and inoculated orthotopically to the fat pads of mammary glands (3 × 10^6^ cells/100 µl PBS containing 50% Matrigel) or intracardially to the left ventricle (1 × 10^6^ cells/50 µl PBS) of 6- to 8-week-old female Fox Chase SCID Beige mice (Charles River, Wilmington, MA). Tumor volume and metastasis were monitored and assessed weekly by bioluminescence imaging (BLI) using an IVIS® Spectrum or Lumina XR Optical Imaging System. A Scanco viva CT 40 system was used to examine skeletal lesions. At the end of the studies, mice were euthanized, and tumor tissues and organs were harvested for histopathological analysis.

### Statistical analysis

All assays were done in triplicates for each of the independent experiments. Differences between groups were analyzed using Student’s *t* test (two groups) or one-way ANOVA (three or more groups). For Kaplan–Meier survival analysis, statistical significance was determined by the log-rank test. Other data were analyzed using GraphPad software (GraphPad Prism version 5.01 for Windows). Results are expressed as the mean ± SD. The *p* value < 0.05 was considered statistically significant.

## Results

### *KRT13 overexpression increases breast cancer proliferation, migration and invasion *in vitro*, and promotes tumorigenesis and metastasis *in vivo

**Fig. 1:**
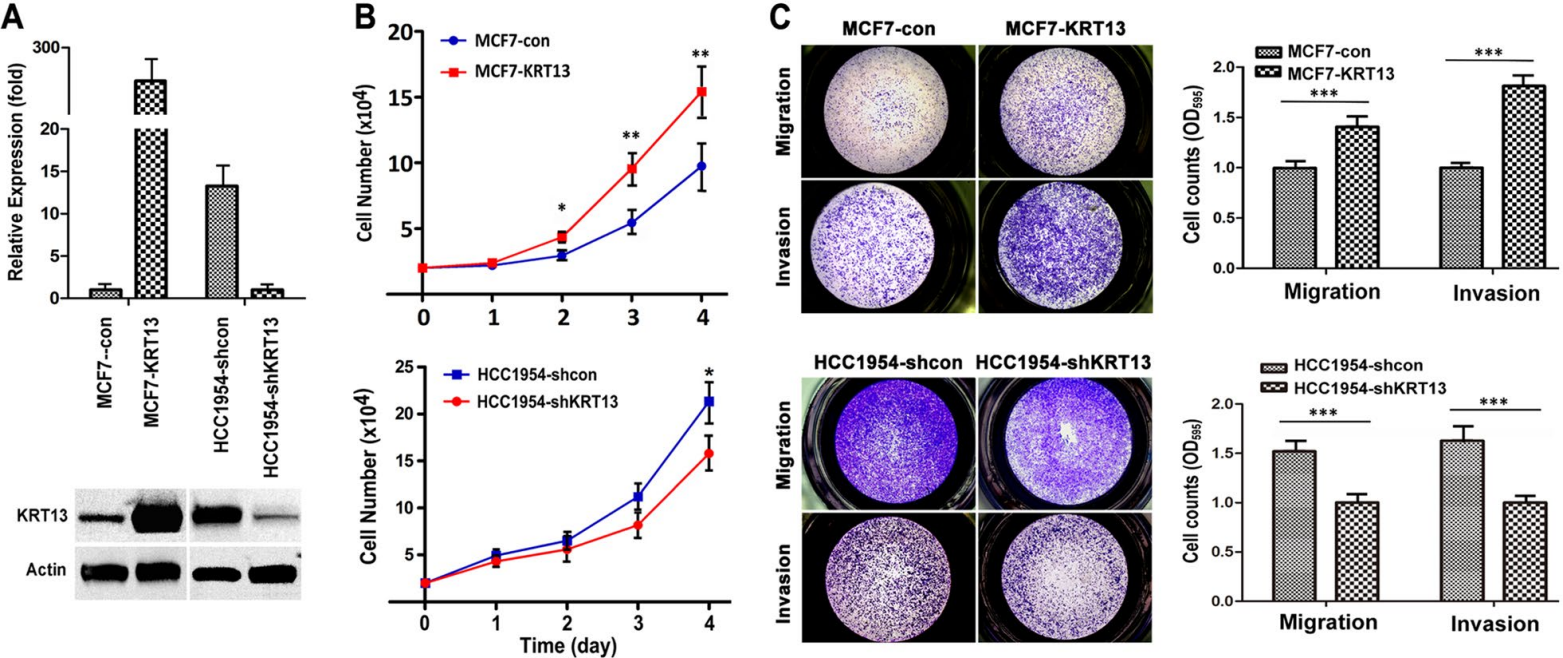
KRT13 overexpression increases MCF7 cell proliferation, migration and invasion. MCF7 cells, which show low endogenous KRT13 expression, were transduced for KRT13 overexpression. HCC1954 cells with high KRT13 level were subjected to KRT13 knockdown. **A** levels of KRT13 in the transduced clones as detected with RT-PCR (upper panel) and western blotting (lower panel). **B** effect of KRT13 on cell proliferation as determined by automatic cell counting. **C** effect of KRT13 on migration and invasion as determined by triplicate transwell assays, which was quantified in graphs (right). For all the presentations, *p ≤ 0.05; **p ≤ 0.01; ***p ≤ 0.001. Each of the assays was repeated at least once and similar results were obtained

**Fig. 2:**
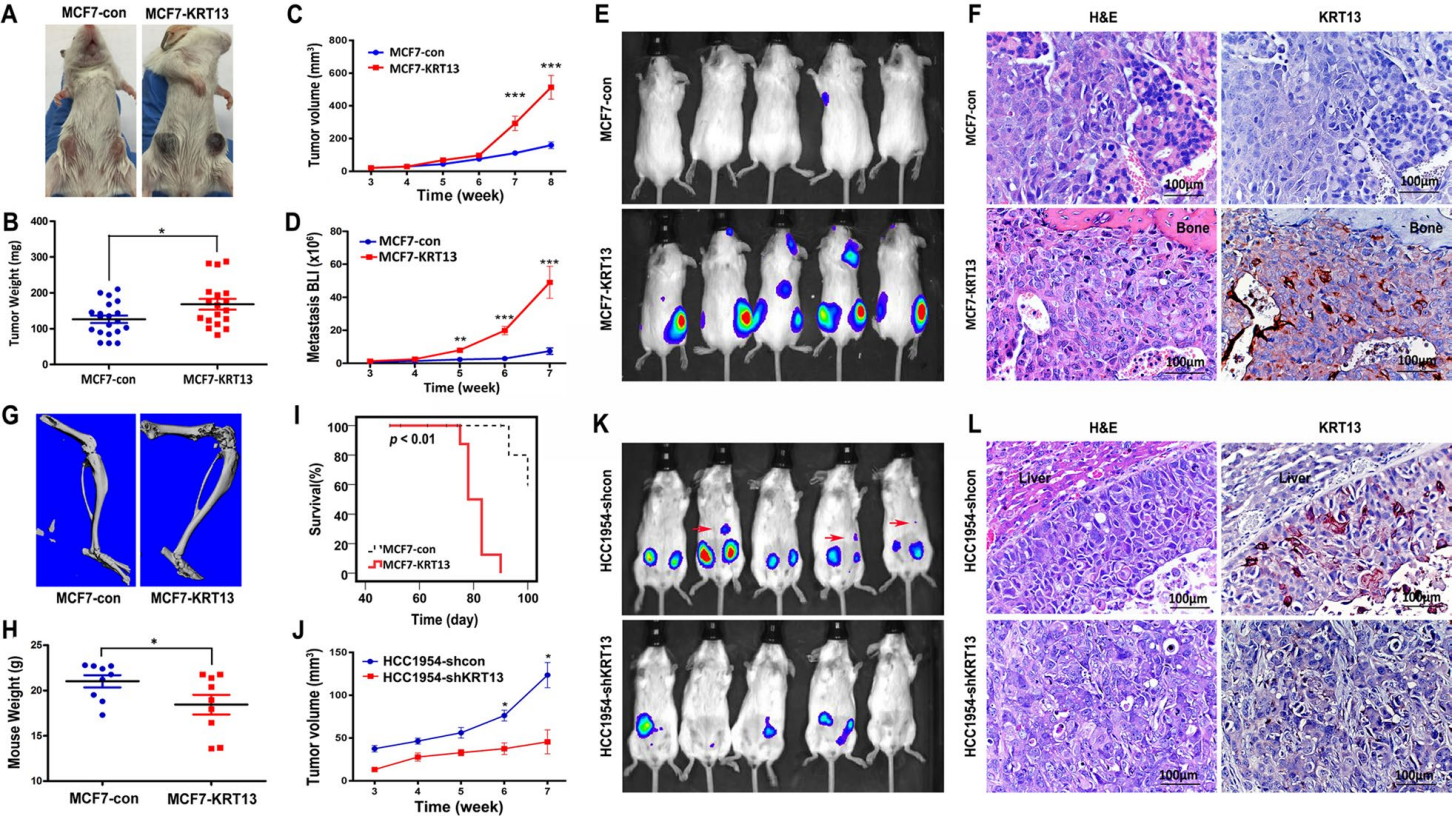
KRT13 induces tumor formation and metastasis of breast cancer cells in mice. **A** MCF7-KRT13 cells were inoculated orthotopically to female SCID mice (10 mice/group, 2 sites/mouse). Representative tumors were photographed 7 weeks after inoculation. **B** orthotopic tumors were resected and weighed 8 weeks after inoculation. **C** weekly changes in tumor volume were calculated with the formula: V (mm^3^) = length (mm) × width^2^ (mm^2^) × 0.5236. **D** following intracardiac inoculation of luciferase-tagged MCF7-KRT13 cells, whole body BLI imaging were used to assess tumor cell spread and colonization. **E** BLI images of 5 mice in each group 7 weeks after intracardiac inoculation. **F** representative H&E stain and KRT13 IHC detection in consecutive sections of MCF7-KRT13 bone tumors (200 ×). **G** a representative µCT image showing osteolytic lesion caused by MCF7-KRT13 tumor by intracardiac inoculation. **H** body weight of the tumor-bearing mice at 8 weeks after intracardiac inoculation. **I** Kaplan–Meier survival plot. **J** HCC1954-shKRT13 cells orthotopically inoculated to mouse breasts (5 mice/group, 2 sites/mouse) displayed reduced tumor incidence (6/10) and tumor size. **K** orthotopic breast tumor metastases were detected with BLI in 3/5 mice (red arrow) at week 8. No metastasis was detected in mice inoculated with HCC1954-shKRT13 cells. **L** representative images of H&E staining and KRT13 IHC detection in orthotopic breast tumor liver metastases at week 8 (200 ×). In the HCC1943-shKRT13 group, a liver metastatic tumor detected 19 weeks after inoculation was used. For all the presentations, *p ≤ 0.05; **p ≤ 0.01; ***p ≤ 0.001

We previously reported that KRT13 induced prostate cancer progression and metastasis [18]. In this study, we investigated the potential role of KRT13 in breast cancer using cell lines that expressed low (MCF7) or high (HCC1954) endogenous KRT13. We established KRT13-overexpressing MCF7 cells (MCF7-KRT13) and knocked down KTR13 levels in HCC1954 cells (HCC1954-shKRT13) (Fig. 1a). MCF7-KRT13 cells displayed increased proliferation (Fig. 1b), migration (Fig. 1c) and invasion (Fig. 1d); while HCC1954-shKRT13 cells showed reduced aggressive behaviors. Moreover, MCF7-KRT13 cells inoculated orthotopically to mouse mammary glands showed accelerated tumor growth, forming hemorrhagic tumors quite different from the control MCF7-con tumors (Fig. 2a), with significantly increased tumor weight (Fig. 2b) and volume (Fig. 2c). When inoculated intracardially, MCF7-KRT13 cells colonized multiple sites (Fig. 2d) leading to tumor formation in lung, liver, adrenal gland, mesenteric lymph nodes, jaw, and other bones (Fig. 2e and Additional file 1: Table S2), which were confirmed by IHC analysis (Fig. 2f). Interestingly, when MCF7-KRT13 cells were cultured in vitro, high levels of KRT13 could be readily detected by western blotting (Fig. 1a); whereas in xenograft tumors, KRT13 overexpression was often seen unevenly by IHC analysis, probably suggesting temporal or spatial modification of the protein in the tumor microenvironment. Bone µCT scans revealed severe osteolytic lesions (Fig. 2g), and the mice had significant weight loss (Fig. 2h) and shortened survival (Fig. 2i).

Similar correlations between KRT13 levels and xenograft tumor formation were observed with HCC1954 cells. HCC1954-shcon xenograft tumors caused prevalent metastases to the liver, adrenal glands and lung as early as 8 weeks post-inoculation. Conversely, HCC1954-shKRT13 cells formed smaller tumors (Fig. 2j) with lower incidence (6/10) (Fig. 2k). KRT13 knockdown delayed HCC1954 tumor metastasis, which was only detected by week 19 (Fig. 2l). These results indicate strongly that KRT13 is associated with elevated aggressive behaviors in breast cancer cells.

### KRT13 overexpression induces epithelial to mesenchymal transition (EMT) and stemness

We established single cell colonies using MCF7-KRT13 cells and detected KRT13 by immunostaining. Interestingly, the staining was stronger at the extending edge than at the center of the colony (Additional file 1: Fig. S1). The edge-positive phenomenon is in accord with our finding that KRT13 is associated with the tumor invasive front in breast cancer tissue specimens (see below).

**Fig. 3:**
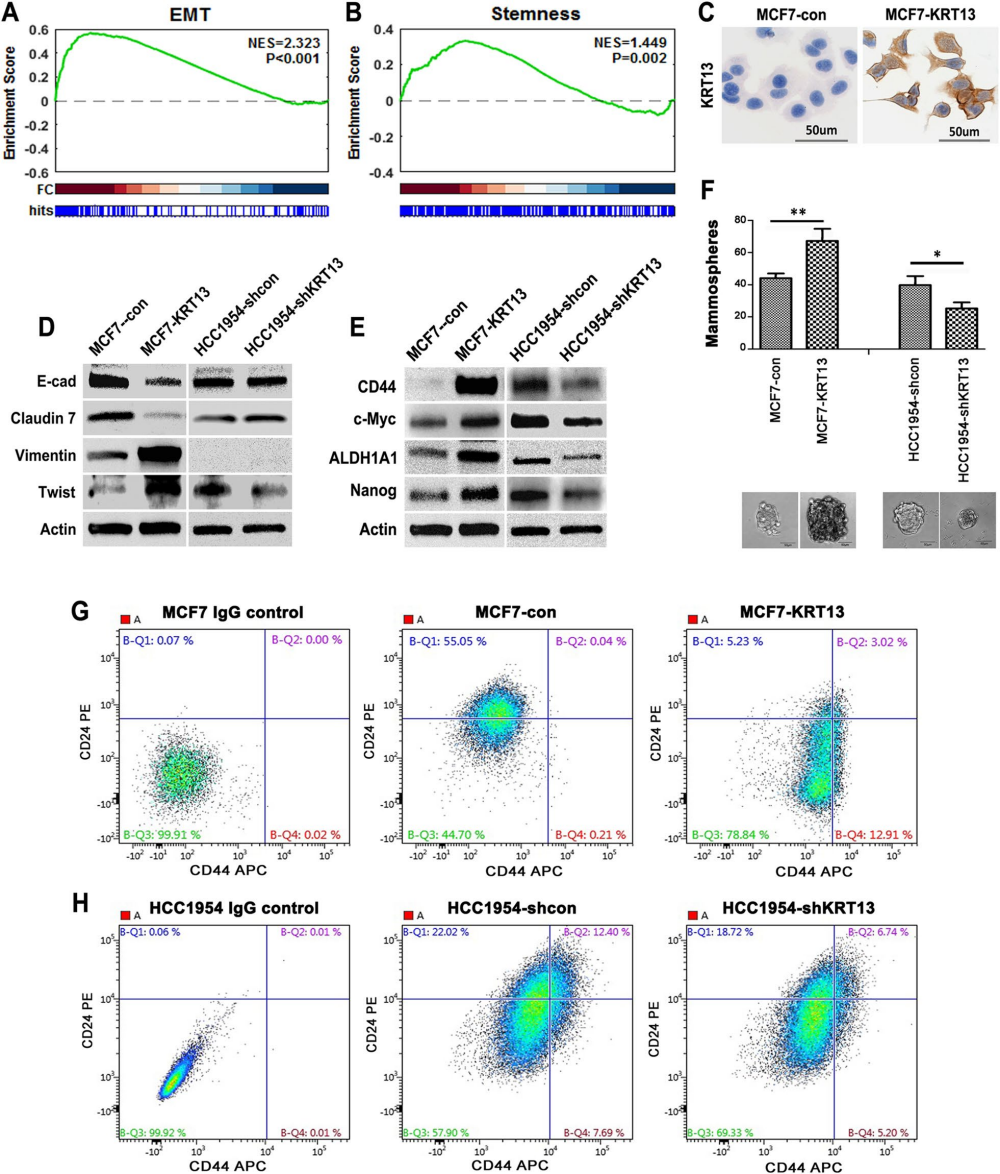
KRT13 overexpression promoted EMT and stemness of breast cancer cells. **A** KRT13 overexpression induced EMT in MCF7 cells as determined by GSEA. **B** KRT13 overexpression induced stemness-related gene expression as determined by GSEA. **C** KRT13 immunostain revealed morphologic changes in MCF7-KRT13 cells (400 ×). **D** altered expression of EMT-associated proteins as determined by western blotting. **E** altered expression of stemness-related proteins. **F** number and size changes in mammosphere formation (*p ≤ 0.05 and **p ≤ 0.01). **G** gain of CD44^+^/CD24^−^ stem cell phenotype in MCF7-KRT13 cells as detected by flow cytometry. **H** reduced CD44^+^/CD24^−^ stem cell marker expression in HCC1954-shKRT13 cells

To investigate the mechanism of KRT13-induced morphologic change and aggressive behavior, we conducted RNA-Seq gene expression profiling with the MCF7-KRT13 cells. Bioinformatic data analysis suggested that KRT13 overexpression had switched the cells from a typical epithelial type to more invasive basal cell-like and Her2-positive subtypes (Additional file 1: Fig. S2) [26]. Gene set enrichment analysis (GSEA) suggested that KRT13-overexpression induces EMT (Fig. 3a) and stemness (Fig. 3b) properties. This explains the marked morphologic changes from connected cobble-stone epithelial MCF7 cells to a less cohesive mesenchymal-like arrangement of the MCF7-KRT13 cells (Fig. 3c and Additional file 1: Fig. S1). MCF7-KRT13 cells were also found with decreased epithelial markers E-cadherin and claudin-7 but with increased mesenchymal markers vimentin and twist. Conversely, claudin-7 were up-regulated and twist was down-regulated in HCC1954-shKRT13 cells (Fig. 3d).

We explored the relationship of KRT13 to stem cell properties. A panel of stemness-related marker and regulator proteins were determined in MCF7-KRT13 and HCC1954-shKRT13 cells. Overexpression of KRT13 increased the expression of CD44, c-Myc, ALDH1A1 and Nanog, whereas knockdown of KRT13 was seen with decreased expression of these proteins (Fig. 3e). MCF7-KRT13 cells displayed a more robust self-renewal capability as reflected by accelerated mammosphere formation and increased mammosphere counts (Fig. 3f). In addition, MCF7-KRT13 cells expressed significantly elevated CD44^+^CD24^−^ stem cell features (Fig. 3g), while HCC1954-shKRT13 cells showed slightly decreased CD44^+^ levels (Fig. 3h). These results suggest that a high KRT13 level promotes stem cell properties.

### KRT13 induces aggressiveness through activation of c-Myc

**Fig. 4:**
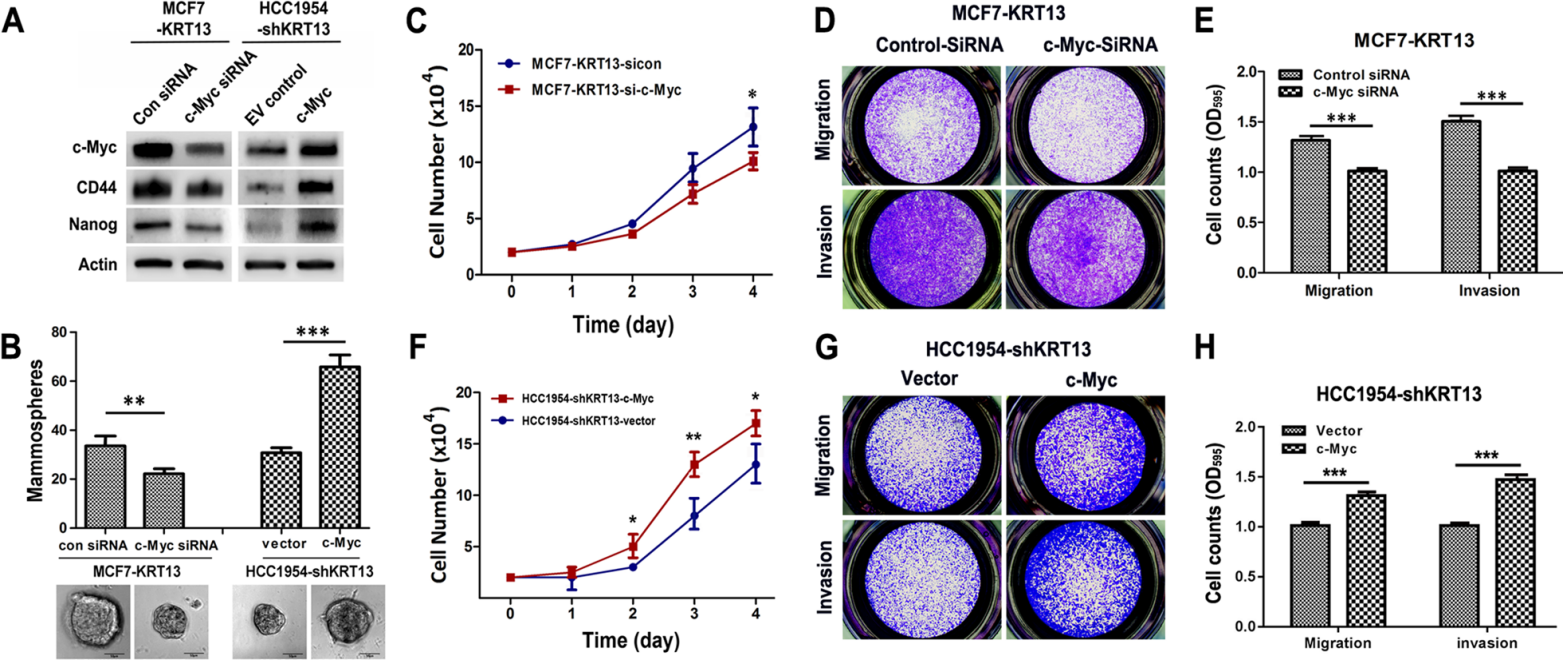
KRT13 induces stem cell properties and malignant progression through c-Myc activation. The expression of c-Myc was molecularly manipulated in KRT13-overexpressing or KRT13-knockdown breast cancer cells. **A** c-Myc silencing in MCF7-KRT13 cells and c-Myc overexpression in HCC1954-shKRT13 cells. Western blot revealed that changes in c-Myc level caused similar changes in CD44 and Nanog proteins. **B** c-Myc silencing inhibited mammosphere formation, while c-Myc overexpression reversed the effect of shKRT13. **C** c-Myc silencing reduced MCF7-KRT13 cell growth. **D** c-Myc silencing inhibited MCF7-KRT13 cell migration and invasion. **E** c-Myc overexpression increased HCC1954-shKRT13 cell growth. **F** c-Myc overexpression increased HCC1954-shKRT13 cell migration and invasion. For all presentations, *p ≤ 0.05; **p ≤ 0.01; ***p ≤ 0.001)

We previously reported that KRT13 overexpression in prostate cancer cells was associated with stemness gene expression, including c-Myc [18]. Here, we manipulated c-Myc expression in KRT13-overexpressing and KRT13-knockdown breast cancer cells. Specific siRNAs effectively repressed c-Myc expression in MCF7-KRT13 cells (Fig. 4a). The c-Myc repression was accompanied by decreased expression of CD44 and Nanog (Fig. 4a), reduced number and size of mammosphere formation (Fig. 4b), cell growth (Fig. 4c), invasion and migration (Fig. 4d). In HCC1954-shKRT13 cells, overexpression of c-Myc induced the expression of CD44 and Nanog (Fig. 4a) and promoted mammosphere formation (Fig. 4b), cell growth (Fig. 4e), invasion and migration (Fig. 4f). Results from these analyses suggest that c-Myc is a key KRT13 downstream mediator promoting aggressive behavior and stem cell-like properties in breast cancer cells.

### KRT13 interacts with PG and decreases its expression and nuclear translocation

**Fig. 5:**
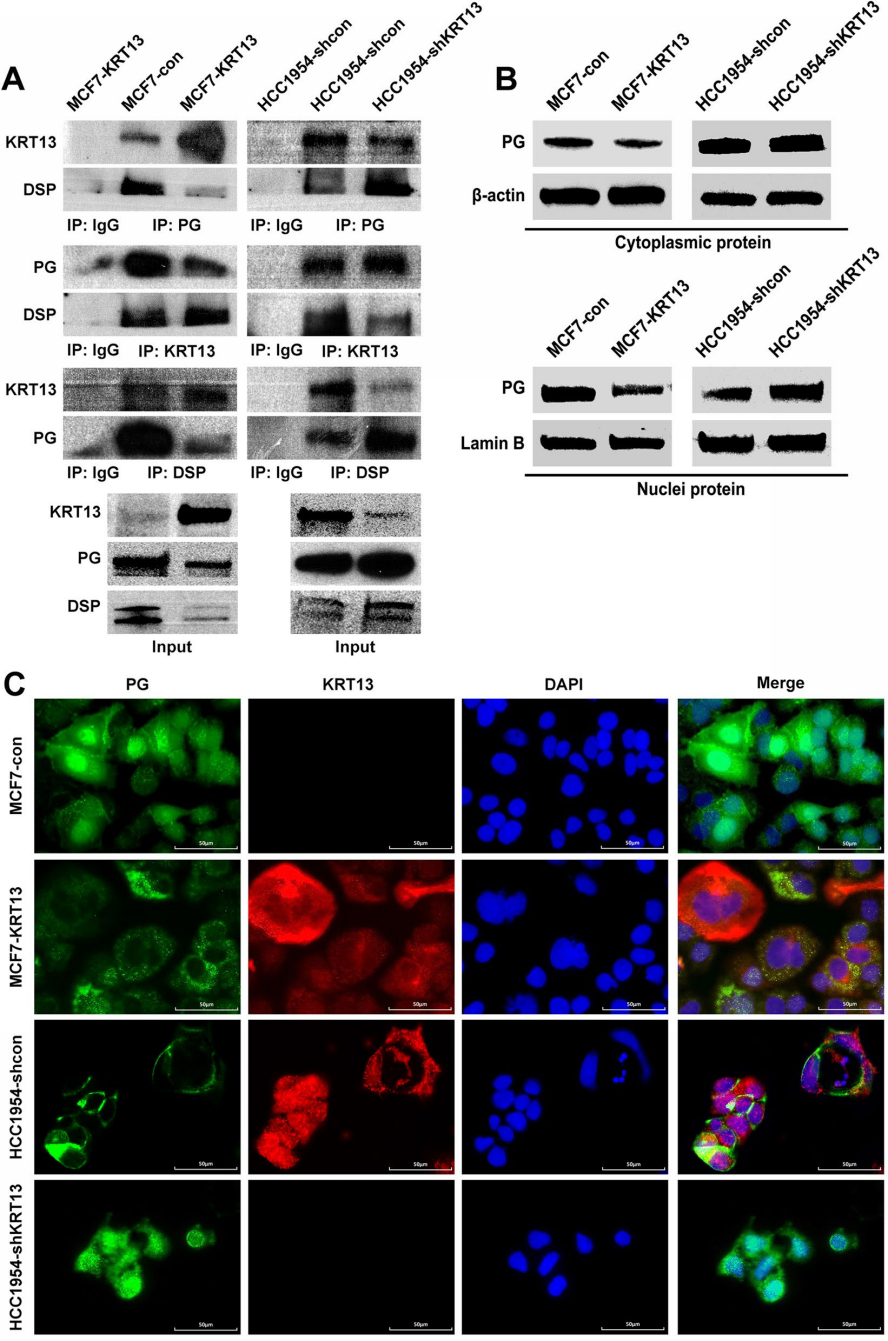
KRT13 suppressed the expression and nuclear translocation of PG by direct interaction. **A** KRT13 interaction with PG and DSP as confirmed by co-IP and western blotting. **B** cytoplasmic and nuclear extracts were subjected to detection for PG protein by western blotting. **C** subcellular localization of KRT13 and PG proteins as detected by immunofluorescence staining

To explore how KRT13 elicits oncogenic signaling in cancer progression and metastasis, we carried out studies to identify intracellular proteins that were interactive to KRT13. In co-IP experiments, antibodies to KRT13 were used in MCF7-KRT13 cells to pull down protein complexes, which were characterized with liquid chromatography-tandem mass spectrometry (LC–MS/MS) and spectral counting methods. Bioinformatic analyses suggested 37 pulled-down proteins that were significantly higher in MCF7-KRT13 cells than MCF7-con cells (Additional file 1: Table S3). We found abundant DSP and PG co-precipitated with KRT13. In co-IP and western blotting analyses with the MCF7-KRT13 and HCC1954-shKRT13 cells, KRT13 interacted directly with both PG and DSP, because either anti-KRT13, anti-PG or anti-DSP antibody was able to pull down all three proteins (Fig. 5a).

Throughout these studies, MCF7-KRT13 cells were found with decreased PG and DSP levels, while HCC1954-shKRT13 cells expressed increased PG and DSP (Fig. 5a). When subcellular distribution was examined, MCF7-KRT13 cells showed significantly reduced cytoplasmic and nuclear PG. In reverse, HCC1954-shKRT13 cells showed significantly increased nuclear PG with a slight increment of PG in the cytoplasm (Fig. 5b). Similar results were detected by immunofluorescence assay (Fig. 5c). KRT13 overexpression seemed to suppress PG and DSP protein levels although the mechanism has yet to be elucidated. Among these cytoskeleton proteins, PG is known to be a potential signaling mediator, translocating to the cell nucleus to exert its tumor suppressor function [27]. Lowered PG levels upon KRT13 overexpression may contribute to the aggressive behavior observed in MCF7-KRT13 cells. These results suggest that KRT13 is capable of binding with the DSP/PG complex, sequestering and lowering the expression and nuclear translocation of PG to promote oncogenic signaling.

### KRT13 expression is correlated with c-Myc levels in clinical breast cancer

**Fig. 6:**
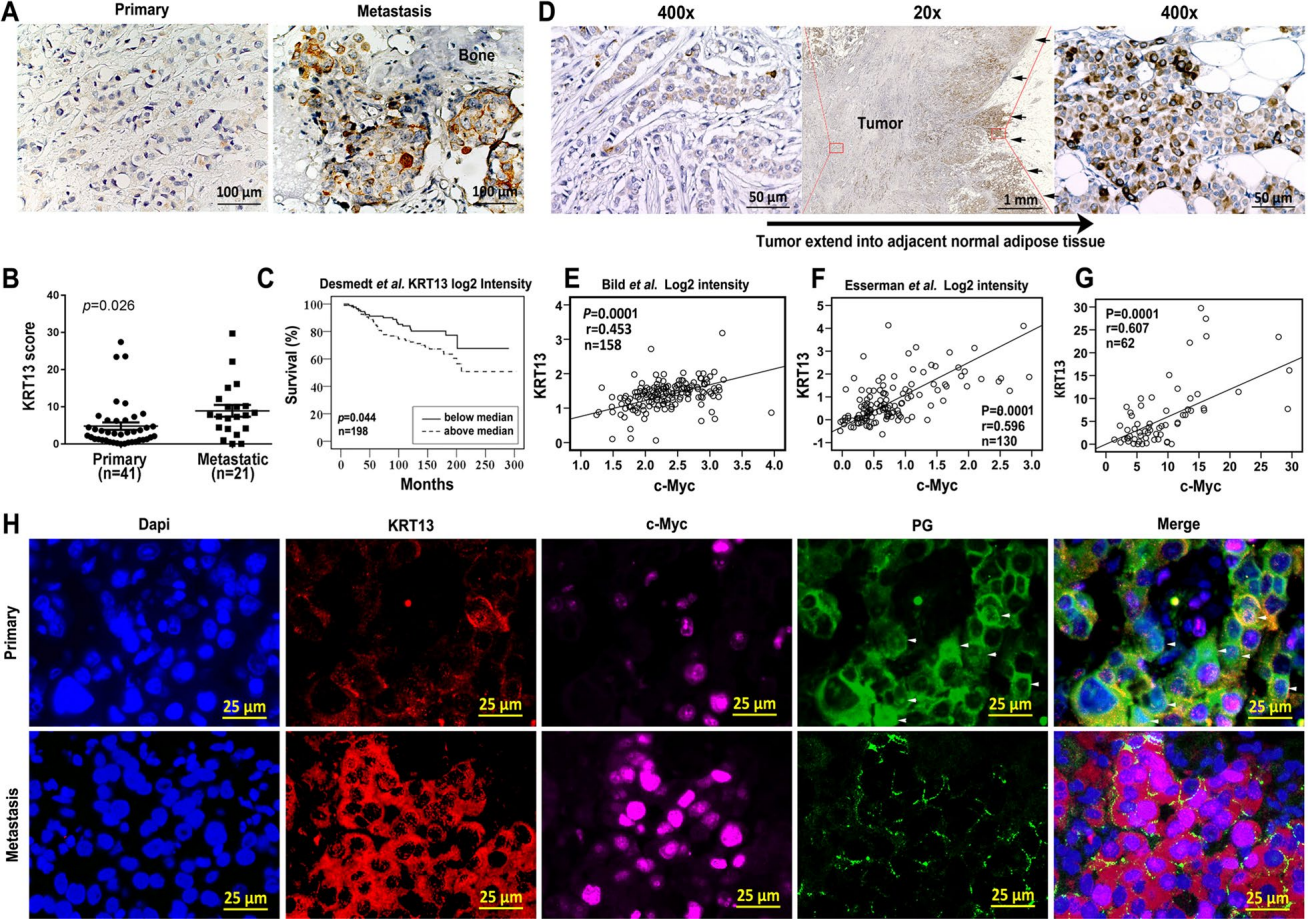
Aberrant KRT13 expression in breast cancer progression and metastasis. Representative results are shown. **A** metastatic breast cancer (N = 21) expressed higher KRT13 than the primary tumor as determined by IHC staining (N = 41). **B** mQDL staining scores. **C** KRT13 expression in primary breast cancer is associated with decreased patient overall survival (Oncomine gene expression datasets). **D** higher levels of KRT13 are expressed at the invasive front in 5/13 primary breast cancer tumors (Arrows). **E**, **F** the relationship of KRT13 and c-Myc expression as suggested by Oncomine analyses. **G** the relationship of KRT13 and c-Myc level in 62 clinical breast tumor specimens as determined by mQDL. **H** representative mQDL analysis suggested that high KRT13 expression in clinical breast tumor metastasis was associated with elevated c-Myc and lowered PG expression

We determined KRT13 expression in 41 primary and 21 metastatic breast cancer tissue specimens by IHC (Fig. 6a). KRT13 expression in metastatic specimens (12/21) was significantly higher than in the primary breast cancer (13/41) (Fig. 6b). In addition, the Oncomine gene expression database (www.oncomine.org) showed that KRT13 expression in primary breast cancer was associated with decreased patient overall survival (Fig. 6c), similar to our report on prostate cancer [18]. KRT13-positive cancer cells were detected at the invasive front of breast cancer (Fig. 6d) as well as prostate cancer (Additional file 1: Fig. S3).

There was a positive correlation between KRT13 and c-Myc expression in this study and in the Oncomine database (Fig. 6e–g). Using three pairs of clinical specimens, we further confirmed the association between KRT13, PG, and c-Myc by mQDL (Fig. 6h), as both KRT13 and c-Myc levels were elevated, whereas both cytosolic and nuclear PG levels were lowered in metastatic tumors than in the primary tumor specimens. A high expression and positive nuclear PG was associated with low KRT13 and c-Myc expression, while low cytoplasmic PG expression was associated with moderate-high KRT13 and c-Myc expression (Fig. 6h and Additional file 1: Fig. S4). These data suggest that KRT13 can downregulate PG expression and reduce its nuclear translocation, abrogate PG-mediated c-Myc suppression, and promote cancer progression.

## Discussion

Breast cancer metastasis is a complex multistep pathologic process. A deeper understanding of the process is critical for improving therapeutic outcomes. KRTs are intermediate filament proteins supporting structural integrity and functions of cells. Recent studies suggest that aberrant expression of KRTs is associated with cancer progression and metastasis. For instance, KRT19 was shown to be a cancer stem cell marker in hepatocellular carcinomas [28], associated with poor overall survival [29]. KRT14 was positive in leader tumor cell clusters which disseminate collectively in breast cancer metastasis [30]. In this study, we identified KRT13 as a novel cancer marker and an oncogenic promoter. Overexpression of KRT13 markedly enhanced breast cancer cell growth, migration, and invasion in vitro (Fig. 1) and promoted tumorigenesis and metastasis in athymic mice (Fig. 2). Conversely, knockdown of KRT13 reduced proliferation and aggressive behaviors (Figs. 1 and 2). These findings unveil for the first time the important role of KRT13 in promoting breast cancer progression and metastasis. The role is particularly relevant to breast cancer treatment, because KRT13 is a target gene of estrogen receptor α activation, while both estrogen and tamoxifen behave as antagonists of the KRT13 gene [4–6].

Recent studies suggested that breast cancer is a stem cell disease, with cancer stem cells critical for cancer dissemination [31, 32]. KRT13 may be associated with stemness because its expression is characteristically localized in suprabasal layers and enriched in prostatic tubule-initiating cells [4–6]. KRT13 plays essential roles in maintaining stem cell homeostasis and symmetric self-renewal in the prostate epithelial layer [33]. In this study, KRT13 induced stem cell phenotypes in MCF7 breast cancer cells (Fig. 3), and increased expression of stemness-related genes of CD44, c-Myc, ALDH1A1 and Nanog. KRT13 promoted the CD44^+^CD24^−^ phenotype, a combination of breast cancer stem cell markers associated with invasion and poor prognosis [34, 35]. KRT13 also induced the formation of mammospheres and increased self-renewal capability. These studies thus suggested KRT13 as a stemness-related mediator contributing to progression and dissemination.

The proto-oncogene c-Myc plays a key role in tumor progression [36, 37]. Its oncogenic activity is associated with poor prognosis [38] and is considered to drive a stem-like phenotype in breast cancer [39]. In our study, KRT13-induced stemness, behavioral changes, and xenograft tumor growth and metastasis could be attributed to c-Myc dysregulation. To prove that KRT13 functions through c-Myc up-regulation, we conducted loss and gain of c-Myc function experiments in KRT13-overexpressing and KRT13-knockdown cells, in which siRNA-mediated c-Myc suppression reversed the stemness and growth advantage, while reduced self-renewal and aggressiveness were compensated by c-Myc overexpression (Fig. 4). These results suggest that KRT13 can up-regulate c-Myc expression leading to stem cell features and aggressiveness.

Stemness and EMT in cancer cells are in tight crosstalk [40, 41]. KRT13-overexpressing MCF7 cells undertook a less cohesive mesenchymal-like shape markedly different from the epithelial-like parental MCF7 cells (Fig. 3). KRT13 overexpression also increased the expression of mesenchymal stromal markers such as vimentin and twist, concomitantly with decreased epithelial markers of E-cadherin and claudin 7. These findings provide evidence that KRT13 activated EMT programs to increase migratory and invasive properties in MCF7 cells.

KRT13 complexed with both DSP and PG to regulate downstream signaling pathways. DSP, an obligate component of desmosomal plaques, connects the desmosomal cadherin/PG/plakophilin (PKP) complex to intermediate filaments. In human lung cancer, DSP was observed to enhance PG expression and act as a tumor suppressor [27]. PG is also a major component of the adherens junctions and desmosomes mediating cell–cell adhesion. Loss of PG reduces cell–cell contact to promote cancer invasion and dissemination [14, 42–44]. In our study, KRT13-overexpression led to decreased DSP and PG, reducing tumor suppressor functions in MCF7-KRT13 cells.

Importantly, PG may be translocated to the nucleus. Both cytoplasmic and nuclear PGs could function to suppress tumor growth and metastasis [14, 45] by modulating the expression of genes involved in stemness, cell-cycle control and cell invasion [16, 17]. The expressions of some important regulatory proteins, such as P53 [15], c-Myc [46], SOX4 [47] and CD133 [48], are all under PG modulation. Nuclear PG is capable of interacting with Tcf/Lef transcription factors to inhibit downstream genes in the Wnt signaling pathway, including c-Myc [49, 50]. In our study, PG expression and nuclear translocation were reduced following KRT13-overexpression (Fig. 5). The reduced PG level may relieve inhibition of c-Myc gene expression, resulting in up-regulated c-Myc driving cancer EMT, stemness and tumor metastasis (Fig. 6). Conversely, KRT13 silencing increased PG expression and nuclear translocation, decreasing EMT, stemness, tumor growth and metastasis. This probability was further strengthened by single cell mQDL staining of clinical breast cancer tissues, which demonstrated that KRT13 expression was inversely correlated with PG, which was inversely correlated with c-Myc (Fig. 6). The molecular mechanisms by which PG regulates c-Myc levels in breast cancer cells need to be investigated further.

We reported previously that forced KRT13 expression drove prostate cancer metastasis through a RANKL-independent mechanism [18], although RANKL-mediated signal network was shown to be associated with EMT, stemness, neuroendocrine or neuromimicry, osteomimicry and tumor cell bone colonization [51]. In this study, we obtained results indicating that KRT13 did not affect RANKL, RANK, or OPG levels (Additional file 1: Fig. S5) in breast cancer cells, suggesting that KRT13-induced breast cancer progression and metastasis may be regulated by a RANKL-independent mechanism. On the other hand, it was surprising that KRT13-overexpression in prostate cancer cells elicited both bone and brain metastases, while no brain dissemination was detected in the breast cancer mouse model in this study, even following intracardiac inoculation. Co-incidentally, the expression of neuromimicry genes including CgA and NSE, elevated in prostate cancer KRT13-overexpresing cells, was not elevated in breast cancer MCF7-KRT13 cells (Additional file 1: Fig. S5). A detailed comparative study of KRT13 function between breast and prostate cancer is warranted.

## Conclusions

In summary, this is the first study reporting that KRT13 interacts with PG/DSP complexes to downregulate the expression and nuclear translocation of PG, which suppresses c-Myc-dependent signaling pathways and consequently inhibits EMT, stem cell-like properties and metastasis. Our findings thus identify potential therapeutic targets for breast cancer progression and metastasis.

## Supplementary Information

**Additional file 1**. Additional file of KRT13 promotes stemness and drives metastasis in breast cancer through a plakoglobin/c-Myc signaling pathway.

## Data Availability

Available upon request to leland.chung@cshs.org.

## Abbreviations

IFs: Intermediate filaments
qRT-PCR: Quantitative real-time polymerase chain reaction
RNA-seq: RNA sequencing
IHC: Immunohistochemical
PBS: Phosphate buffered saline
co-IP: Co-immunoprecipitation
GSEA: Gene set enrichment analysis
mQDL: Multiplexed quantum dot labeling
EMT: Mesenchymal transition
