# Supplementary material for: KRT13 promotes stemness and drives metastasis in breast cancer through a plakoglobin/c-Myc signaling pathway
Source: Breast Cancer Res. 2022 Jan 25;24:7. doi: 10.1186/s13058-022-01502-6 (PMC8788068; doi:10.1186/s13058-022-01502-6)
Supplement: Supplementary file 1 — Additional file 1. Additional file of KRT13 promotes stemness and drives metastasis in breast cancer through a plakoglobin/c-Myc signaling pathway. [file 13058_2022_1502_MOESM1_ESM.docx]

**Supplementary data**

| **Table S1.** Primer sets used in this study. | | |
| --- | --- | --- |
| **Genes** |  | **Real-time PCR primer sequences** |
| KRT13 | F: | 5’-AGGACGCCAAGATGATTGGTT-3’ |
|  | R: | 5’-GTGGTAACAGAGGTGCTACGG-3’ |
| ALDH1A1 | F: | 5’-AGTGCCCCTTTGGTGGATTC-3’ |
|  | R: | 5’-AAGAGCTTCTCTCCACTCTTG-3’ |
| c-Myc | F: | 5’-TCAAGAGGCGAACACACAAC-3’ |
|  | R: | 5’-GGCCTTTTCATTGTTTTCCA-3’, |
| Nanog | F: | 5’-ATGCCTCACACGGAGACTGT-3’ |
|  | R: | 5’-AAGTGGGTTGTTTGCCTTTG-3’ |
| CD44 | F: | 5’-TCAGAGGAGTAGGAGAGAGGAAAC-3’ |
|  | R: | 5’-GAAAAGTCAAAGTAACAATAACAGTGG-3’ |
| N-cadherin | F: | 5’-GGTGGAGGAGAAGAAGACCAG-3’ |
|  | R: | 5’-GGCATCAGGCTCCACAGT-3’ |
| E-cadherin | F: | 5’-CCCGGGACAACGTTTATTAC-3’ |
|  | R: | 5’-GCTGGCTCAAGTCAAAGTCC-3’ |
| claudin-7 | F: | 5’-AGGAGAGAGCACTTTGGACAG-3’ |
|  | R: | 5’-CACTCACCTGAACCGGAAGT-3’ |
| Vimentin | F: | 5’-GGAAGAGAACTTTGCCGTTGAA-3’ |
|  | R: | 5’-GTGACGAGCCATTTCCTCCTT-3’ |
| SOX-2 | F: | 5’-TTGCTGCCTCTTTAAGACTAGGA-3’ |
|  | R: | 5’-CTGGGGCTCAAACTTCTCTC-3’ |
| RANKL | F: | 5’-TCCCATCTGGTTCCCATAAA-3’ |
|  | R: | 5’-GGTGCTTCCTCCTTTCATCA-3’ |
| CgA | F: | 5’-CCCCACTGTAGTGCTGAACC-3’ |
|  | R: | 5’-GGAGTGCTCCTGTTCTCCC-3’ |
| SYP | F: | 5’-CTCGGCTTTGTGAAGGTGCT-3’ |
|  | R: | 5’-CTGAGGTCACTCTCGGTCTTG-3’ |
| β‐actin | F: | 5’-TTGTTACAGGAAGTCCCTTGCC-3’ |
|  | R: | 5’-ATGCTATCACCTCCCCTGTGTG-3’ |

**Table S2.** Increased metastatic incidence in mice intracardially inoculated with MCF7-KRT13 cells (**p* ≤ 0.01).

| Metastasis site | MCF7-con (N = 8) | MCF7-KRT13 (N = 8) |
| --- | --- | --- |
| Bone | 2/8 | 8/8* |
| Abdominal tissues | 1/8 | 3/8 |
| Lung | 0/8 | 2/8 |

**Table S3.** Mass spectrometry showed 37 proteins, after co-immunoprecipitation by KRT13 antibody, that were significantly higher in MCF7-KRT13 cells than MCF7-con cells.

| **No.** | **Protein name** | **Gene symbol** |
| --- | --- | --- |
| 1 | Keratin, type I cytoskeletal 9 | KRT9 |
| 2 | Keratin, type II cytoskeletal 8 | KRT8 |
| 3 | Keratin, type I cytoskeletal 18 | KRT18 |
| 4 | Keratin, type II cytoskeletal 7 | KRT7 |
| 5 | Keratin, type I cytoskeletal 16 | KRT16 |
| 6 | Keratin, type II cytoskeletal 6B | KRT6B |
| 7 | Keratin, type II cytoskeletal 1 | KRT1 |
| 8 | Keratin, type I cytoskeletal 19 | KRT19 |
| 9 | Keratin, type I cytoskeletal 14 | KRT14 |
| 10 | Desmoplakin | DSP |
| 11 | DNA-dependent protein kinase catalytic subunit | PRKDC |
| 12 | T-complex protein 1 subunit alpha | TCP1 |
| 13 | Vimentin | VIM |
| 14 | Heat shock cognate 71 kDa protein | HSPA8 |
| 15 | Tubulin beta-4B chain;Tubulin beta-4A chain | TUBB4B;TUBB4A |
| 16 | GTP-binding nuclear protein Ran | RAN |
| 17 | Prelamin-A/C;Lamin-A/C | LMNA |
| 18 | 40S ribosomal protein S3 | RPS3 |
| 19 | Tubulin alpha-1B chain | TUBA1B |
| 20 | Putative elongation factor 1-alpha-like 3;Elongation factor 1-alpha 1 | EEF1A1P5;EEF1A1 |
| 21 | Heat shock protein beta-1 | HSPB1 |
| 22 | ATP synthase subunit alpha, mitochondrial | ATP5A1 |
| 23 | Keratin, type II cytoskeletal 6A | KRT6A |
| 24 | Junction plakoglobin | JUP |
| 25 | 60S ribosomal protein L13a | RPL13A |
| 26 | ATP-dependent RNA helicase A | DHX9 |
| 27 | 40S ribosomal protein S11 | RPS11 |
| 28 | 40S ribosomal protein S13 | RPS13 |
| 29 | 60S ribosomal protein L7a | RPL7A |
| 30 | 60S ribosomal protein L11 | RPL11 |
| 31 | Prohibitin-2 | PHB2 |
| 32 | 60S ribosomal protein L3 | RPL3 |
| 33 | 60S ribosomal protein L28 | RPL28 |
| 34 | Keratin, type I cytoskeletal 17 | KRT17 |
| 35 | ATP-binding cassette sub-family D member 3 | ABCD3 |
| 36 | Nucleoporin NUP188 homolog | NUP188 |
| 37 | RNA-binding protein 14 | RBM14 |

**Figure S1.** Single cell MCF7-con and MCF7-KRT13 colonies were subjected to immunocytochemical staining with antibodies to KRT13. KRT13 was strongly expressed at the extending edge of the MCF7-KRT13 cells (100×).

**
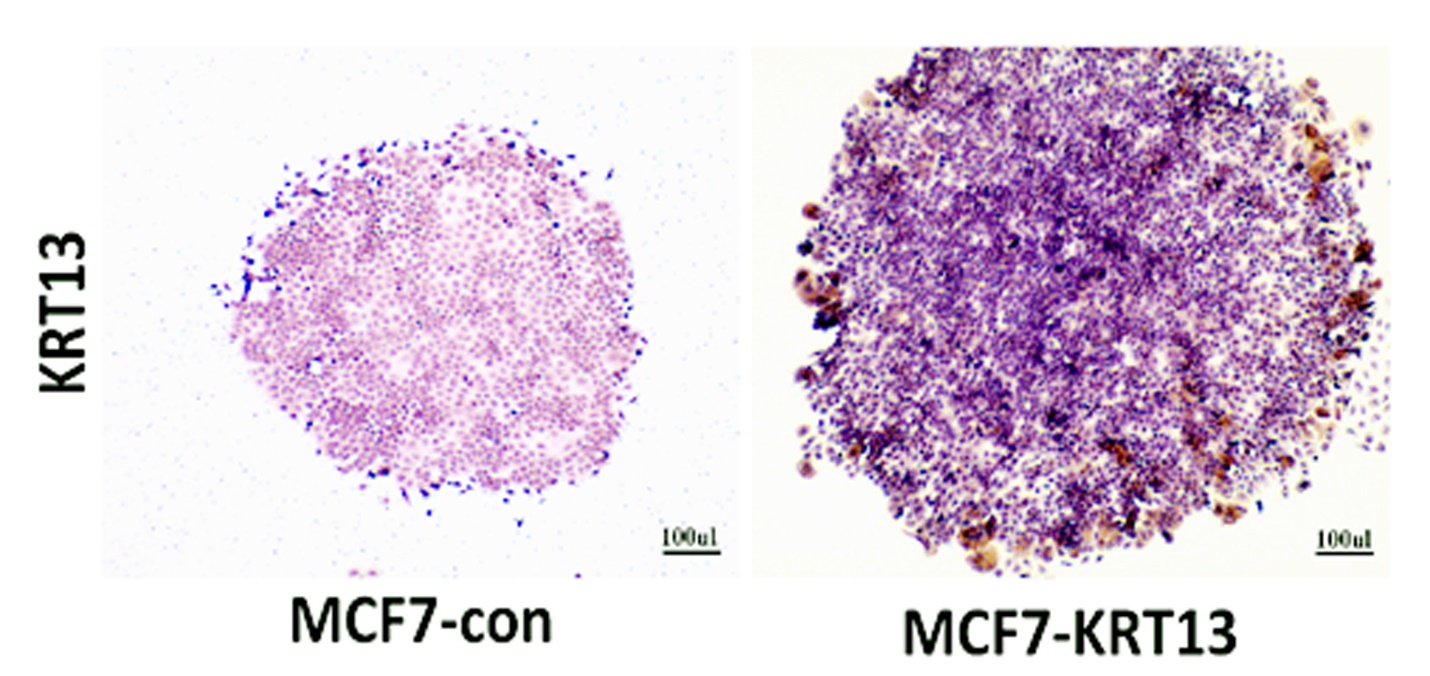
**

**Figure S2.** Heatmap analysis of RNA-seq gene expression profiles shows that MCF7-KRT13 cells are co-clustered with the Basal and Her2 breast cancer subtypes, compared to MCF7-con cells which are co-clustered with the Luminal A subtype.

**
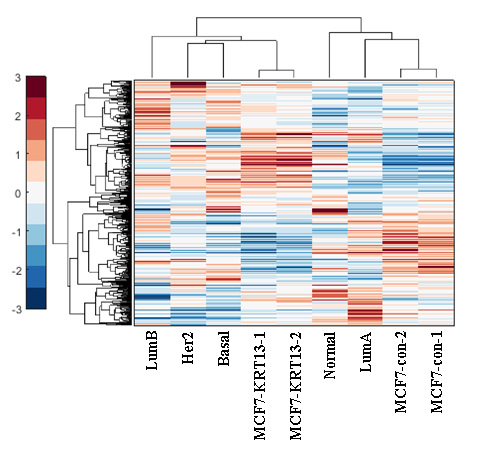
**

**Figure S3.** Clinical primary prostate cancer specimens were subjected to KRT13 IHC. KRT13-positive cancer cells were detected at the invasive front of the tumor (arrows). Image magnifications from left to right: 400×, 40× and 400×.

**
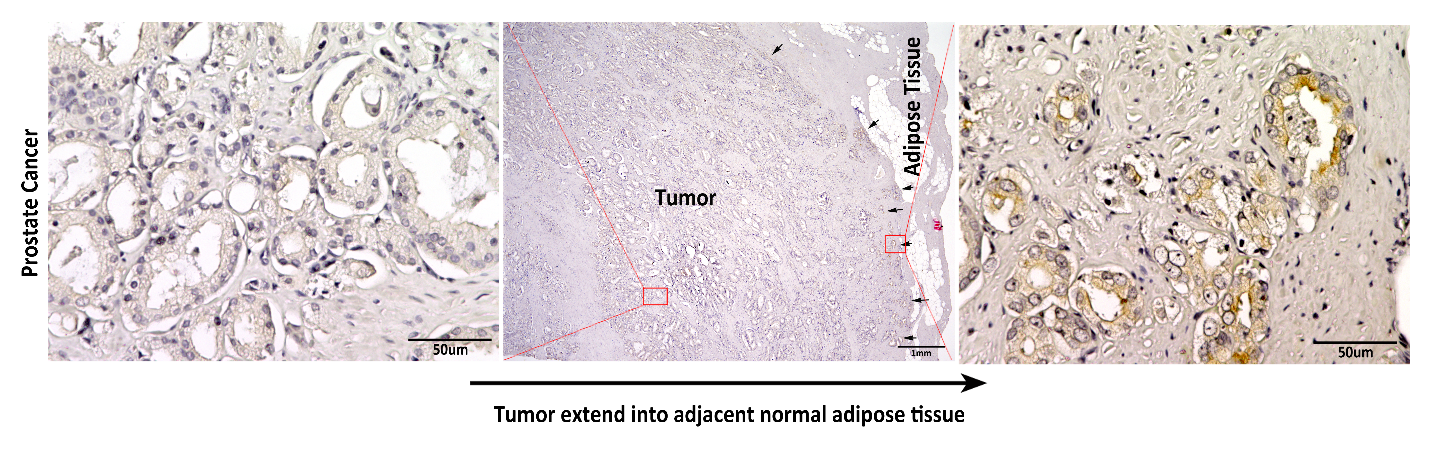
**

**Figure S4.** Co-expression of KRT13, PG and c-Myc on the single cell level by mQDL analysis in breast cancer tissues. Protein expression intensity was determined with inForm software and analyzed by Spearman Correlation Analysis.

**

**

**Figure S5.** KRT13 overexpression does not elevate neuroendocrine/neuromimicry or osteomimicry biomarkers in breast cancer cells as detected with western blotting analysis.

**

**
